# Transformation of Waste Glasses in Hydroxide Solution

**DOI:** 10.3390/ma18245565

**Published:** 2025-12-11

**Authors:** Przemysław Czapik, Katarzyna Borek

**Affiliations:** Faculty of Civil Engineering and Architecture, Kielce University of Technology, Al. Tysiąclecia Państwa Polskiego 7, 25-314 Kielce, Poland; k.komisarczyk@tu.kielce.pl

**Keywords:** waste glass, pozzolanic reaction, XRD, SEM, thermal analysis, calcium hydroxide

## Abstract

Unused glass waste represents a potentially valuable secondary raw material for the production of construction materials. This study aimed to investigate the chemical and structural transformations occurring in soda-lime container glasses of different chemical compositions when exposed to alkaline environments. Such alkaline conditions are characteristic of processes involved in the production of lime–sand materials or Portland cement-based composites, where they are essential for the occurrence of pozzolanic reactions. The investigation was conducted on powders derived from three types of container glass differing in color, which were stored in Ca(OH)_2_ and NaOH solutions. The samples were analysed using X-ray diffraction (XRD), differential thermal and thermogravimetric analysis (DTA–TG), and scanning electron microscopy (SEM). The results confirmed that all tested glasses exhibited pozzolanic reactivity, although differences were observed in the composition of the reaction products and the kinetics of the transformation processes. A deeper understanding of these differences may contribute to more effective utilization of waste glass as a raw material in the manufacturing of construction materials.

## 1. Introduction

Ceramic materials, which include building materials, brick (clinker and stoneware), mineral binders such as Portland cement, autoclaved building materials, i.e., sand lime products, and autoclaved aerated concrete, are the basic and most widely used materials of modern construction. Since the presence of SiO_2_ is predominant in the chemical composition of these materials, raw materials containing significant amounts of this oxide [1,2,3,4] are required for their production. However, silica can exist in various forms, both on its own and in combination with water and various metal cations, forming numerous compounds. Due to the form of silica in the potential raw material, its suitability for the production of building materials varies. For this reason, the search is still on for the most cost-effective raw materials as a source of silica for the ceramics industry.

In ceramic technologies, building materials are obtained by thermal treatment (firing) or hydrothermal treatment (autoclaving) of the raw material mass. These processes result in phase transformations, during which SiO_2_ can form phases that provide the resulting materials with the desired characteristics. It is known that the less stable a phase is, the more easily it can undergo chemical reactions and the less energy is required for such a reaction to take place. Thus, the processing of the raw material, which includes the more reactive phases, can be simpler and less energy-intensive and can therefore be carried out at lower temperatures.

From a thermodynamic point of view [1], the state of matter characterised by very low stability is the glassy state, in which structural equilibrium is not achieved. Potentially, any substance can be carried out into the glassy state, but compounds with a mesodesmic structure, meaning one in which cations occur that compensate for exactly half of the existing charge, allowing for the formation of a cation-anion-cation bridge exhibit a particular ease of conversion into this state. Examples of such compounds are silicates, in which the Si-O bond valence is 1, and Si-O-Si bridges are formed [1]. Responsible for this is the presence of SiO_2_, which is the best-described glass-forming oxide. According to Zachariasen’s classical division, oxides are divided into glass-forming, glass-phase modifiers and intermediate oxides on the basis of their ability to form a glass bond. Silicon dioxide is the basic component of silicate glasses, which are the most common inorganic glasses. Glasses consisting only of amorphous silica, possibly containing only a slight addition of other oxides, are called quartz glasses. In industrial practice, the most common glazes produced are those comprising, in addition to SiO_2_, other glass-forming, modifying and intermediate oxides, the proportion of which, however, usually does not exceed 50 per cent by weight [2,3]. In the spaces between the SiO_4_ tetrahedra, modifying oxide cations are located, which, by breaking the Si-O-Si bridges, modify the silica glass bond [1]. These primarily include alkali metal oxides, alkaline earth oxides and some transition metal oxides. Cations of intermediate oxides as well as other glass-forming oxides can substitute tetrahedral silicon to co-form the bond of silicate glass and thus influence its characteristics.

As SiO_2_ content in glass rises, its chemical resistance improves [2], making it advantageous to use glasses with altered structures for raw material applications. For instance, soda-lime glass, one of the most commonly produced types, contains sodium oxide (Na_2_O) added deliberately to lower melting, softening points, and viscosity, enhancing manufacturability. However, this reduces its chemical resistance, which could be an advantage for reuse purposes. When water contacts such glass, it adsorbs onto the surface, bonding structurally and forming OH groups that cover it. These groups promote further water absorption by thickening the water film. In the presence of alkali, water penetrates deeper, causing leaching of alkali metal silicates and creating a concentrated hydroxide layer on the surface, potentially leading to visible clouding. Conversely, calcium (CaO) in soda-lime glass enhances chemical resistance by decreasing hygroscopicity.

When temperatures exceed 60 °C, water can significantly dissolve silica. In such conditions, water’s impact on glass extends beyond leaching to include dissolving the entire material. The dissolution process releases alkaline products, which can further deteriorate the glass. These alkaline compounds act by completely dissolving the glass surface layer. During this process, stable anions like Si_2_O_52_-, SiO_44_-, and SiO_32_- form from the silica. Meanwhile, the glass’s cations generate hydroxides or aluminate-like compounds. Because silica in glass is thermally unstable and more soluble than in crystalline form, it is particularly vulnerable to these processes. At 25 °C, amorphous silica solubility ranges from 39 to 120 mg/L, whereas quartz solubility is only 2.9 mg/L [5,6].

Soda-lime glass has found wide use as a packaging material. Their production is the largest segment of the glass industry [7,8]. Its chemical composition results from balancing desired physico-chemical properties with the need for optimal melting, clarification, and molding conditions. Although adding sodium can sometimes compromise quality, it remains a common practice [2]. The main features of packaging glasses, such as color and transparency, depend on their chemical makeup. Producing colorless glass requires higher-quality raw materials with limited coloring oxides. Conversely, colored glasses often use lower-quality raw materials with intentionally added coloring oxides, which may influence other properties of the final product [8].

The desired color in glass is typically achieved by introducing transition metal ions into its structure [2,3]. To produce brown glass, Fe_2_O_3_, e.g., as Venetian red or (polishing) rouge, is used alongside sulfur and carbon as reducing agents; alternatively, iron oxides may be introduced via various waste materials, such as pyrite cinder, or iron-rich, easily melting rocks. Fe_3_O_4_ imparts a green color to the glass. A bright green hue is commonly obtained using chromium compounds, such as Cr_2_O_3_ and particularly K_2_Cr_2_O_7_, which readily melts and dissolves in glass; other chromium sources include Na_2_Cr_3_O_7_, K_2_CrO_4_, or Na_2_CrO_4_∙10H_2_O. Furthermore, to achieve a purer shade of chromium-colored glass, copper is added, and the batch is melted under oxidizing conditions. Other oxides that also color the glass green include V_2_O_3_ and Pr_2_O_3_.

In the production of colorless glass, decolorizers are also used to remove the tint originating from coloring oxides, especially iron, introduced with the raw materials. There are many types of decolorizers, with MnO_2_ being among the most popular.

The production of container glass commonly uses cullet, which is a simple way of recycling glass [8]. Today, cullet is used to produce 90% of glass packaging. However, the amount of cullet that can be used for this purpose depends on the type of glass being produced. In the case of clear glass, produced with the highest-quality raw materials, cullet can reach up to 20%. When producing colored glasses, a greater addition of cullet is used, in the case of brown glass up to 50% and in the case of green glass up to 80%. The quality requirements for cullet increase with the demands on the final products and as the proportion of recycled cullet increases. This is important because cullet obtained from public container collections is mostly contaminated. Moreover, it is a mixture of different glasses and, unlike traditional glass raw materials, the chemical composition of cullet can fluctuate considerably. The presence of impurities in the form of metals, ceramics, or rocks can disrupt the structure of the glass and raise its melting temperature. The lack of cullet homogeneity can also affect the clarity and color of the packaging produced. For these reasons, the cullet must undergo a technically complex treatment process before it can be used to make glass again. Therefore, the complete recycling of glass in the manufacturing process is not cost-effective. Unrecycled glass is landfilled [9], which is not an environmentally friendly solution, as it is not biodegradable. Traditionally, glass from multi-colored packaging is recycled the least. The need to manage it opens the possibility of using waste glass in other industries.

In the construction industry, the use of waste glass as a material for various building materials, especially concrete, is being considered. The use of waste glass in concrete production is most often associated with its use as a mineral additive in cement and concrete [10,11,12,13,14,15,16,17]. This solution has the environmental advantage that, in addition to recycling glass, it also makes it possible to reduce the consumption of Portland cement clinker and cement, respectively. This is important because the production of cement clinker, the basic ingredient of cement, results in significant CO_2_ emissions into the atmosphere [4,12,16,17].

Finely ground glass, in the form of glass powder as an additive to concrete, can be classified as a type II additive [18], i.e., a chemically active additive whose presence contributes to the concrete’s strength. This property of glass is closely related to the previously described corrosion mechanism of glass in the presence of water. When there is a high concentration of Ca^2−^ ions in the water, this effect is greatly intensified and a pozzolanic reaction takes place, resulting in the formation of hydrated calcium silicates–the C-S-H phase, which is the primary product of cement setting [4,19]. This is a crucial reaction in cement and concrete technology regarding the use of mineral admixtures. Pozzolanicity is defined as a material’s capacity to react with calcium hydroxide, which forms as a byproduct during cement hydration. This reaction yields hydrated calcium silicates, known as the C-S-H phase, similar to those produced during cement hydration. The formation of this additional C-S-H phase contributes to increased density, strength, and durability of the hydrated cement paste. An important factor for the pozzolanic reaction is the specific surface area and particle size (fineness) of the material undergoing it. If the material’s particle size is too coarse, the pozzolanic reaction may not occur. Additionally, if the surrounding environment contains sodium and potassium hydroxides in addition to calcium hydroxide, an alkali-silica reaction may occur instead of the pozzolanic reaction [12,16,17,20]. The pore solution filling the pores in the cement matrix is rich in various dissolved hydroxides, including calcium, sodium, and potassium hydroxides [4,21], thereby providing the conditions for both the pozzolanic reaction and the alkali-silica reaction to take place. During the alkali-silica reaction, an alkali gel with expansive properties may form, leading to cracks in the concrete and ultimately reducing its durability. It has been confirmed that the alkali-silica reaction can occur when glass cullet grains with a size of 1–2 mm are introduced into the concrete [14,20,22,23]. The same can happen if the fine grains of mineral additives containing amorphous silica agglomerate [24]. For this reason, borosilicate glass is used as a reactive-aggregate standard in testing binder reactivity [25,26].

The pozzolanic reaction accelerates exponentially with increasing temperature. This is because, according to the Arrhenius equation, the solubility of silica increases with increasing temperature [27]. The effect of temperature interaction in turn increases with humidity, as the thermal conductivity of the system then increases [28]. These phenomena have been used in the technology of autoclaved materials, silicate bricks, and autoclaved aerated concrete. The maturation of these materials takes place in special reactors, autoclaves, which maintain a sufficiently high temperature by surrounding them with steam under considerable pressure. Under such hydrothermal conditions, the solubility of crystalline silica in the form of quartz is similar to that of amorphous silica under natural conditions. In this way, silica introduced into the silicate mass in the form of quartz sand can react with the binder, lime or Portland cement, and hydrated lime, forming hydrated lime silicates, such as tobermorite and xonotlite, thereby bonding the entire raw material mass.

Attempts are often made to introduce various materials into the raw material mix for autoclaved materials, as substitutes for sand and, sometimes, for the binder [29]. This is usually dictated by the desire to dispose of waste materials from other industries, but it is also intended to improve the performance of the product after autoclaving. Various studies have examined the use of waste glass as a substitute for quartz sand in autoclaved building products. In addition to the objective of waste management, such action aims to improve production and the properties of the products obtained. Because the solubility of amorphous silica is more than three times that of glassy silica under autoclaving conditions [28], the efficiency of this process can be increased, potentially reducing the time or temperature of production and translating into lower production costs. Previous studies have also shown that replacing quartz sand with glass cullet increases the strength of the autoclaved product while decreasing its weight [30,31]. The observed decrease in mass is linked to the lower density of the glass compared to quartz sand. This factor is important from the point of view of logistics, the delivery of the aggregate and the finished product, as well as the fact that the finished product can carry a higher load while exerting a lower load itself.

In the literature, studies regarding the application of waste glass in building material production are abundant. Most research focuses on mixed waste streams, utilizing multi-colored glass cullet [11]. Less common, however, is research using more homogeneous waste, consisting of glass of a single color [32]. Studies comparing results obtained with different-colored glasses have indicated that the glass color may influence the properties of the resulting building materials [31,33]. However, such studies usually focus on identifying the impact on the material technological properties, such as strength or density, and concentrate on practical application issues.

Nonetheless, detailed basic research in this field remains limited. This paper reports on foundational studies of powders derived from three calcium-sodium glasses with similar chemical compositions and varying colors. The objective was to assess whether these minor differences in composition that affect the coloration have an influence on the course of the pozzolanic reaction, which was tracked by observing phase transformations. X-ray diffraction was employed, supplemented by quantitative analysis using the thermal analysis method, allowing us to determine which phases formed and disappeared, as well as their periods of occurrence. Additionally, microstructure analysis was performed on mature samples. The findings from this study are intended to provide a better explanation, in the future, of the effects of using glass cullet in building materials.

## 2. Materials

The chemical activity of the glass was tested on powders obtained by grinding three cullet glass obtained from color-differentiated packaging. Table 1 shows their chemical composition, determined by WDS X-ray spectrometry in Axios (PANalytical, Almelo, The Netherlands) in accordance with ISO 29581-2:2010 [34] and PN-EN 196-2:2013-11 [35]. These glasses have previously been used in studies on calcium–silicate product properties [36]. As expected, the main differences are in the levels of coloring oxides. In both colored glasses, Fe_2_O_3_ content is significantly higher than in the colorless glass. This oxide gives a reddish hue and contributes to the color of brown glass. Although green glass has a similar Fe_2_O_3_ level, its color is mainly due to the presence of Cr_2_O_3_ [12]. The variation in K_2_O content is noteworthy. It is smallest in colorless glass and largest in green glass. This is linked to the fact that chromium is popularly introduced into glass in potassium compounds [2]. Analyzing Table 1, it can be concluded that in the colored glasses, the coloring oxides and potassium oxide are primarily replaced by CaO and SO_3,_ and possibly Al_2_O_3_, which is more abundant in the colorless glass.

It can be simultaneously concluded that a decolorizer in the form of manganese oxide was not used in the production of the colorless glass.

A significantly higher MgO content is evident in the brown glass compared to the other two glasses. It makes them less chemically resistant [2].

**Figure 1:**
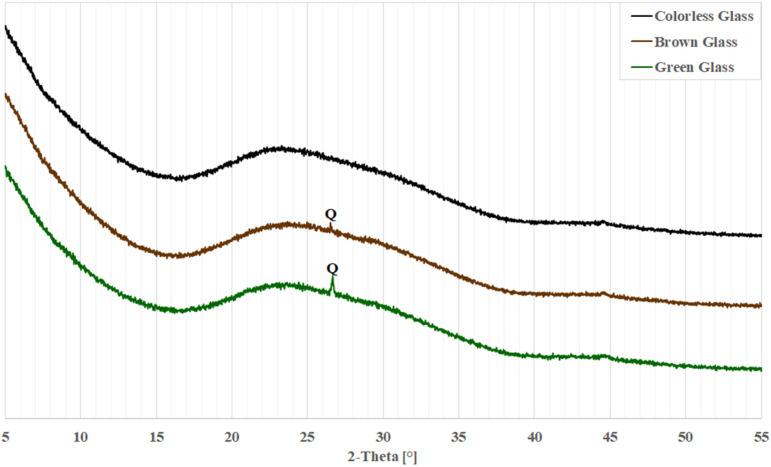
X-ray patterns of waste glass powders. Q—peak from quartz.

Figure 1 shows the diffraction patterns obtained for each glass. For each glass, there is a clear lifting of the background in the 2θ angle range from 17 to 38°, which is related to the amorphous nature of the material under study [9,37,38,39]. In addition, for the brown and green glass, a peak extending beyond the background is visible at 26.65° 2θ, consistent with the main peak from quartz [9,40].

Calcium hydroxide (PA grade) and 1 M NaOH solution were also used in the study.

## 3. Experimental and Methods of Characterization

The chemical activity of the glasses was investigated on the assumption that they could exhibit pozzolanic properties [41]. Sample preparation was divided into two stages. First, waste glass of various colors was crushed in a mortar to obtain a powder with particle size less than 0.063 mm. In the second stage, the powder obtained was mixed with Ca(OH)_2_ in a 3:1 ratio, then placed in sealed polyethylene containers, poured over with a 1 M NaOH solution, and mixed. The prepared suspensions were stored at room temperature. The necessary amount of material was taken each time before the test. The reaction was stopped by adding acetone at the following intervals: 1 h and after 7, 28, 56 and 330 days in order to understand the changes occurring in their phase composition and microstructure.

Phase composition studied by XRD powder diffraction (DSH–Deby-Scherrer-Hull) on an Empyrean diffractometer (PANalytical, Almelo, The Netherlands). Prior to testing, the collected samples were ground by hand in an agate mortar and dried in a stream of cold air. Tests were carried out over the 2θ range of 5–60° at a recording rate of 0.0005° 2θ/s. The resulting diffraction patterns were analyzed using the International Centre for Diffraction Data (ICDD) PDF-2 database 2022 (2022, the International Centre for Diffraction Data, Newtown Square, PA, USA). After 28 days, samples were also collected for phase-composition testing by DTA-TG thermal analysis on a TGA/DSC Q600 analyzer (TA Instruments, New Castle, DE, USA). To do this, the samples were dried at 60 °C under a nitrogen stream for 30 min, after which they were heated to 1000 °C at 10 °C/min. The microstructure and elemental composition of the resulting products were studied, in turn, on samples taken after 330 days using a Quanta FEG 250 scanning electron microscope (SEM) (FEI Company, Hillsboro, OR, USA) equipped with an energy-dispersive X-ray microanalyzer (EDS). The study was carried out under low-vacuum conditions, with electron beam imaging at 5 kV.

## 4. Results and Discussion

### 4.1. Phase Analysis (XRD)

**Figure 2:**
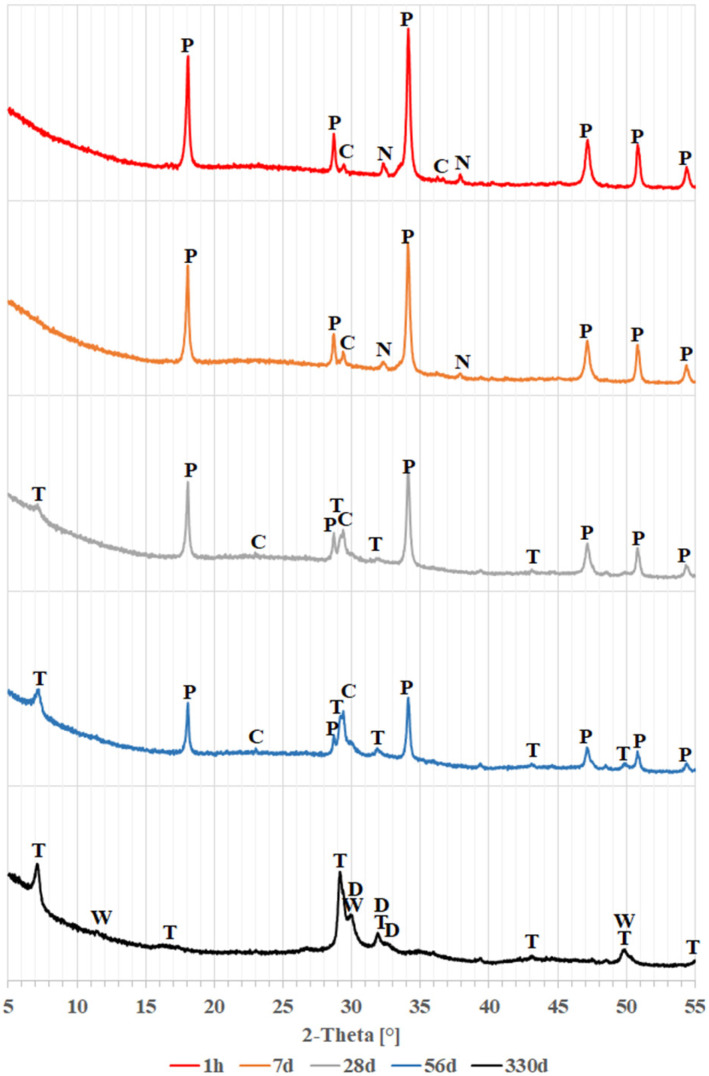
X-ray patterns of colorless glass powder: C—calcite, D—dellaite, N—thermonatrite, T—tobermorite, P—portlandite, W—wollastonite.

**Figure 3:**
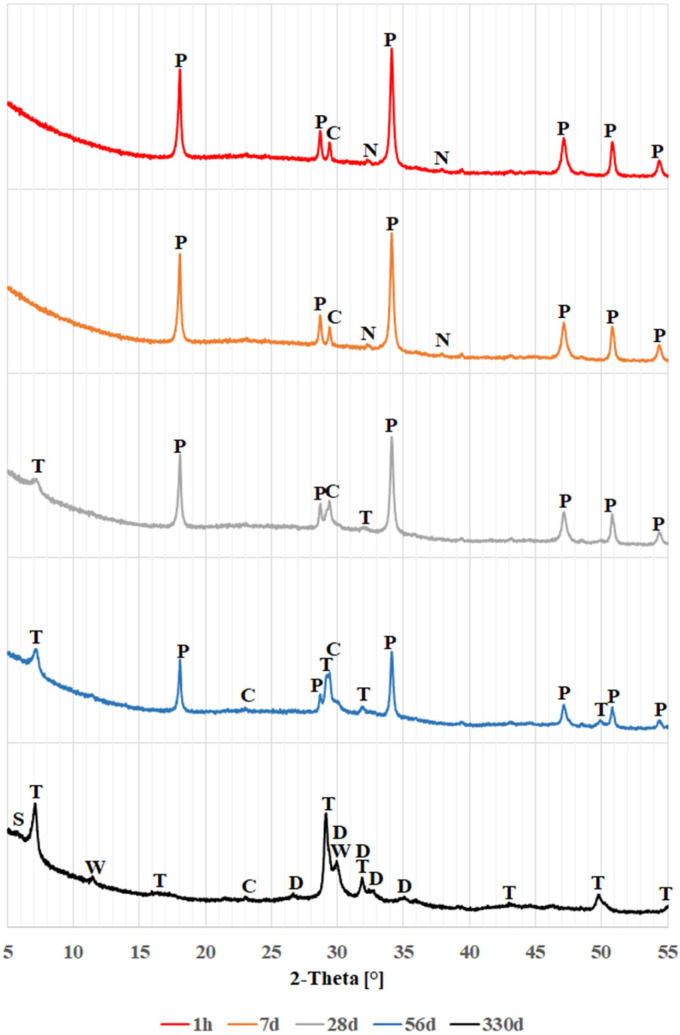
X-ray patterns of brown glass powder: C—calcite; D—dellaite; N—thermonatrite; T—tobermorite; P—portlandite; S—saponite; W—wollastonite.

**Figure 4:**
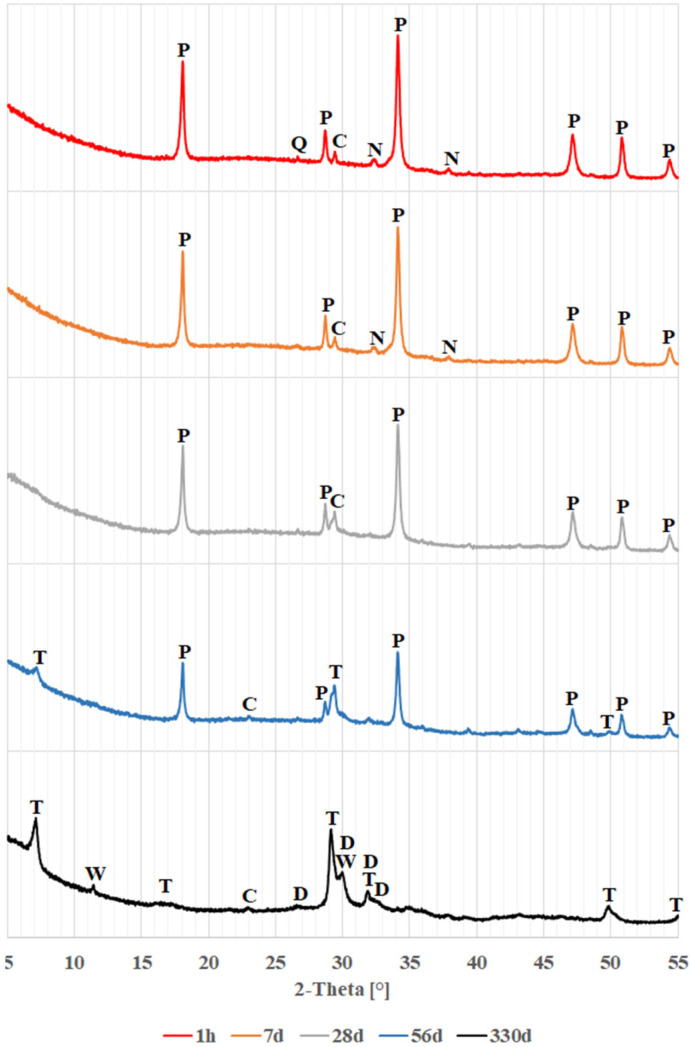
X-ray patterns of green glass powder: C—calcite; D—dellaite; N—thermonatrite; T—tobermorite; P—portlandite; Q—quartz; W—wollastonite.

The results of the study of changes in the phase composition of glass powders exposed to a calcium and sodium hydroxide solution are shown in Figure 2, Figure 3 and Figure 4. These figures show successively the changes occurring for colorless, brown and green glass over a period of 330 days. For legibility, diffraction patterns covering a range of 2θ angles from 5° to 55° are shown here.

In the initial period, up to day 7, the diffraction patterns for all glasses look similar. Primarily visible are peaks from portlandite (calcium hydroxide, Ca(OH)_2_), accompanied by small peaks from calcite (calcium carbonate, CaCO_3_) and thermonatrite (hydrated sodium carbonate, Na_2_CO_3_∙H_2_O). The presence of such peaks can be explained by the fact that calcium hydroxide is the compound added during sample preparation, and both carbonates may have been formed by carbonation of calcium hydroxide and sodium hydroxide, respectively. In particular, the samples may have been exposed to carbonation during the drying process between suspension sampling and examination in the diffractometer. The diffraction pattern of the sample with green glass shows a slight quartz peak at 26.65° 2θ, consistent with the patterns shown in Figure 1.

When colorless and brown glass powders are tested, significant changes in their diffraction patterns are observed after 28 days. From this date onwards, the peaks from the calcium hydroxide decrease significantly, until they disappear completely in the diffraction patterns obtained after 330 days. In place of the disappearing portlandite, peaks identified with hydrated calcium silicates–the C-S-H phase, with a structure similar to tobermorite (Ca_5_Si_6_O_17_∙5H_2_O)–are beginning to become visible. This indicates that a pozzolanic reaction is occurring between the glass and calcium hydroxide [4,9]. After 28 days, peaks from thermonatrite are no longer detectable. It can be concluded that thermonatrite is a transitional phase, formed in the suspensions studied as a result of excess sodium dissolved in solution before the actual reaction of hydroxides with glass begins. Thus, Ibrahim and Meawad’s thesis [42] that thermonatrite formation may contribute to reducing the alkali-silica reaction by immobilizing some of the available sodium may be considered overly optimistic. Following the disappearance of thermonatrite after 28 days, the sodium previously present in it may once again become available for other reactions. It could contribute to the formation of the alkali-silica gel.

The main calcite peak for angle 2θ = 29.50° from day 28 onwards begins to be absorbed by the tobermorite peak occurring for angle 2θ = 29.27°, and after 330 days, it can no longer be distinguished. After 56 days, however, a subordinate calcite peak at 2θ = 23.96° becomes visible, as explained later in this paper.

In the case of green glass, peaks from tobermorite do not appear until 56 days, indicating a slower rate of transformation. In this case, the peaks from the thermonatrite also disappeared, as early as day 28.

The diffraction patterns obtained after 330 days demonstrate the further progression of the pozzolanic reaction. The disappearance of the presence of portlandite is accompanied by an increase in tobermorite peaks and the formation of further calcium and silicon compounds with a dellaite-like structure (Ca_6_Si_3_O_11_(OH)_2_) and saponite-like (Ca0.5(Mg,Fe)_3_((Si,Al.)4O_10_)(OH)_2_∙nH_2_O). El Fami et al. [43], in a similar study of belite hydration in hydroxide solution, also found the formation of dellaite next to tobermorite at a later stage of the reaction. Similarly, Garbev et al. [44] identified dellaite as a product of the reaction of colloidal silica with a solution of calcium and sodium hydroxides occurring at elevated temperatures, encountered, e.g., in deep boreholes [45]. In the case of the peak observed for an angle of 30.10° 2θ attributed to dellaite, it may be suspected that its considerable intensity is the result of overlapping peaks from another phase. Such a phase could be wollastonite (CaSiO_3_), which shows the most intense peak in this area. Also, the peak at 11.50° θ could confirm its presence; however, it should be noted that wollastonite is usually formed at high temperatures [44,46], although there are reports of its growth under laboratory conditions [47]. The presence of dellaite-like phases and possibly wollastonite may indicate that phases with a lower degree of hydration than tobermorite formed in the samples over time.

Only in the brown glass were faint reflections detected, which could indicate the presence of saponite-like structures, possibly related to the high magnesium and iron content of this glass (Table 1).

**Figure 5:**
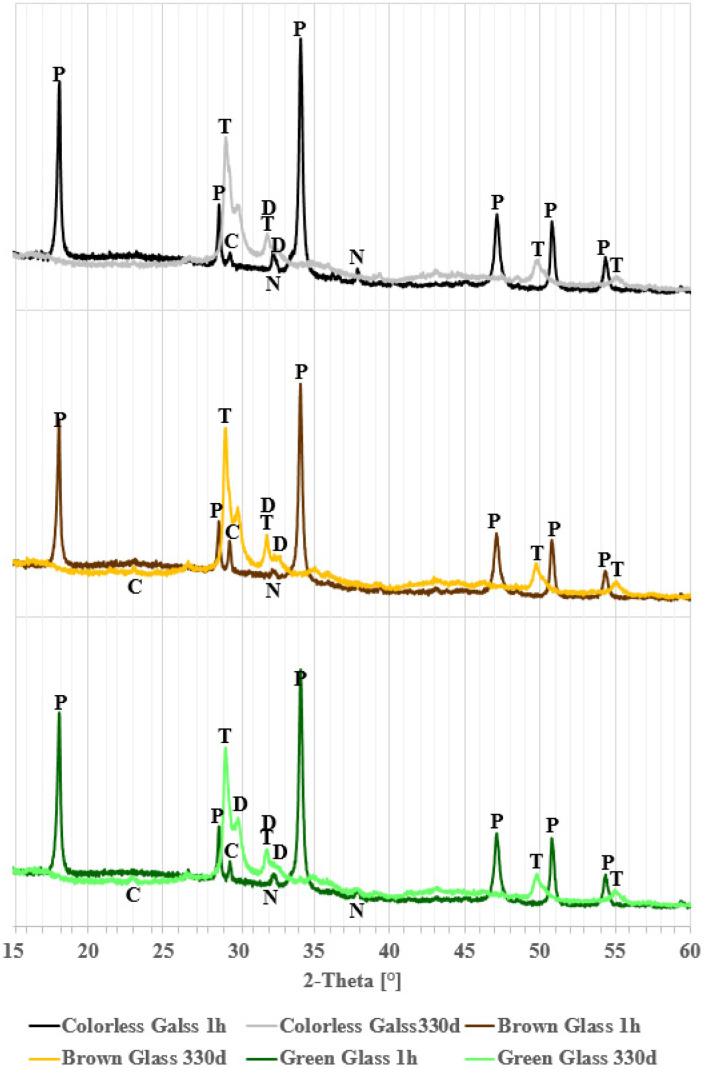
Comparison of X-ray patterns for glass powders stored 1 h and 330 days in hydroxide solution.

Figure 5 compares diffraction patterns in the 2θ range of 15 to 60° for clear, brown, and green glass over the course of one hour to 330 days of reaction. Besides the peaks noted earlier, changes in background levels are observed. The increased background suggests the presence of amorphous phases like glass or amorphous C-S-H [4]. In the figure, fresh samples after 1 h show a raised background between 17 and 37° 2θ, typical of glass [36,37,38,39], as depicted in Figure 1. As the glass reacts with the solution, this background level decreases, revealing the previously hidden calcite reflection at 23.96° 2θ. Concurrently, the background increases from 25 to 38° and 41 to 52° 2θ, indicating formation of amorphous products. The minor calcite reflection implies that this phase is likely not involved in the ongoing chemical reactions.

Table 2 summarizes the phases present in the colorless, brown, and green glass samples as a function of time.

### 4.2. Phase Analysis (DTA–TG)

**Figure 6:**
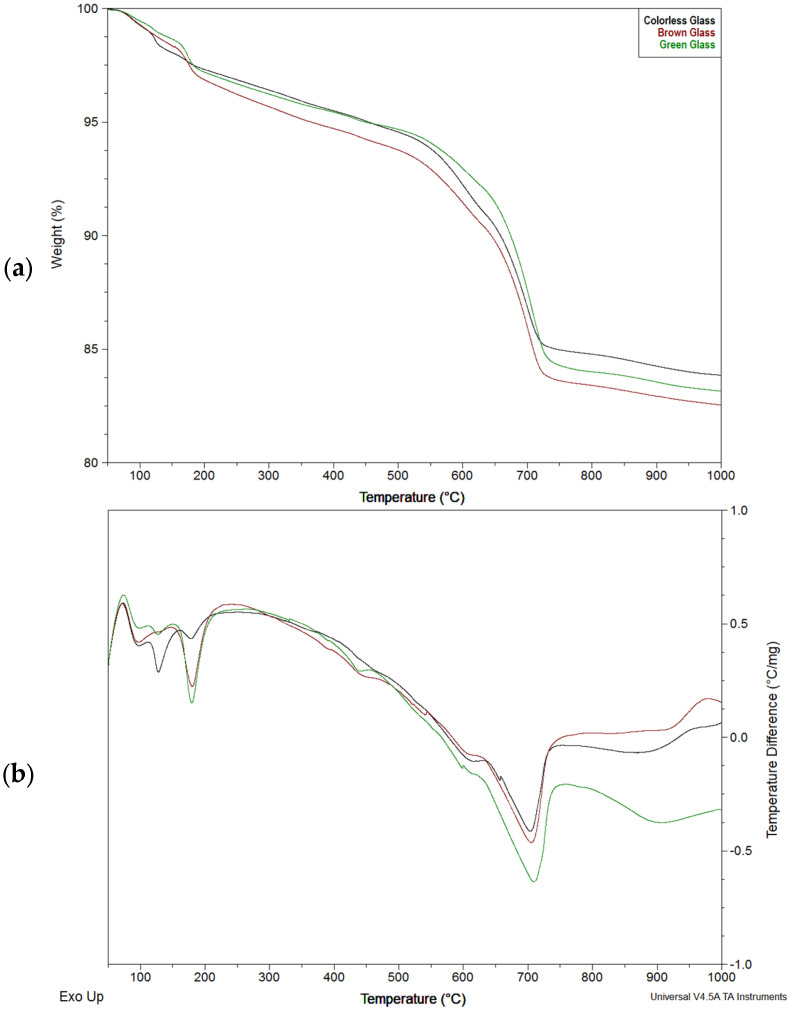
Thermal analysis of glass powder samples stored for 28 days in hydroxide solution: (**a**) TG, (**b**) DTA.

Figure 6 shows the results of thermal analyses for individual glasses after 28 days of reacting with hydroxide solution. This period coincides with the XRD analysis, which showed a difference in the tobermorite formation rate between the green glass and the others. The graphs reveal notable endothermic changes linked to mass loss at temperatures between 50 and 200 °C and between 480 and 770 °C. The first range relates to water loss, with the DTA diagrams indicating three stages. Initially, physically bound water is lost by 100 °C. Then, two endothermic peaks at 125 °C and 180 °C are observed, corresponding to water loss from hydrated calcium silicates [2,40]. This implies two water-binding phases in the study. Interestingly, in clear glass, the transformation peaks at 125 °C, indicating a greater change, while in both colored glasses, the more intense effect peaks at 180 °C. For brown glass, the transformation at 125 °C is barely noticeable.

The transformations associated with mass loss above 480 °C can be explained by the decarbonation of carbonates [45], i.e., calcite detected by X-ray analysis, and the presence of partially carbonated hydrated calcium silicates. It can be seen that mass loss between 480 and 770 °C occurs at two rates. Up to 630 °C, it proceeds more slowly, after which it accelerates and ends around 770 °C, which may indicate the presence of various carbon dioxide-containing phases.

The graphs show minor variations around 440 °C, likely related to calcium hydroxide presence.

The data in the graph are provided in Table 3. The TG results align with the DTA findings. Analysis shows that, due to the reactions occurring, colored glasses—especially brown glass—may have retained more water in their reaction products than colorless glass. This is most evident in a product with a higher decomposition temperature (180 °C), where green glass contained the most bound water. A relatively small change associated with the presence of Ca(OH)_2_ was detected, indicating its presence at just over 2%. This can be explained by the significant indications from the calcium carbonate that resulted from the carbonation of the hydroxide. This can be ascertained by comparing the results of the thermal analysis with those of the X-ray analysis. Figure 2 and Figure 3 show more portlandite and less calcite. As indicated in Table 2, the amount of hydroxide needed to produce the observed carbonate can be several times the remaining portlandite. The DTA curve and the sum of portlandite amounts from verses 4 and 6 in Table 2 may indicate that the greatest amount of portlandite may have remained in the green glass, which may be another proof that the pozzolanic reaction is slower where calcium hydroxide is consumed. Nonetheless, some calcite was present in the samples, formed before sampling and testing, meaning the portlandite amount from verse 6 exceeds the actual amount carbonated.

### 4.3. Microstructure Analysis (SEM-EDS)

**Figure 7:**
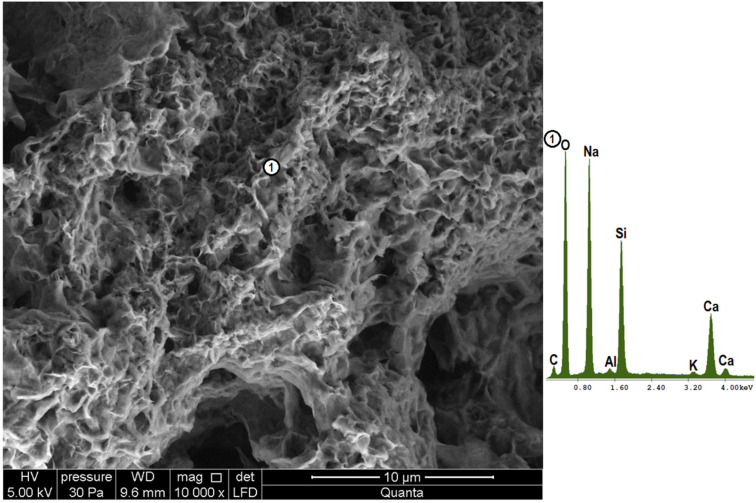
Microstructure of clear glass powder after 330 days of storage in hydroxide solution (magnification 10,000×), together with the elemental composition analysis at point 1.

**Figure 8:**
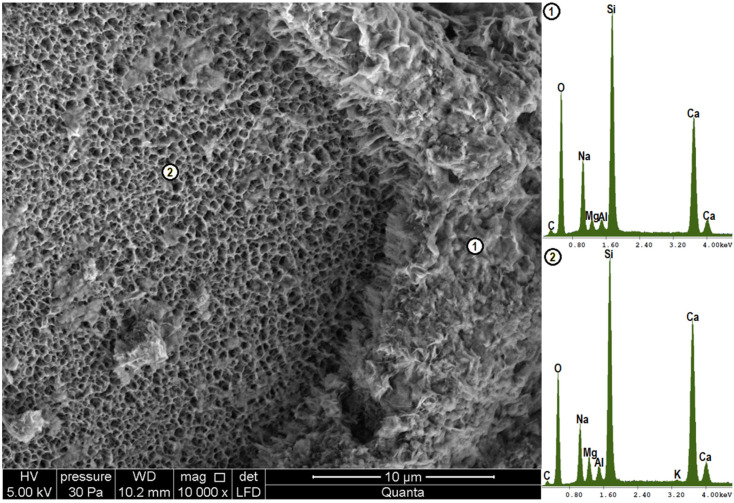
Microstructure of brown glass powder after 330 days of storage in hydroxide solution (magnification 10,000×), together with analysis of elemental composition at points 1 and 2.

**Figure 9:**
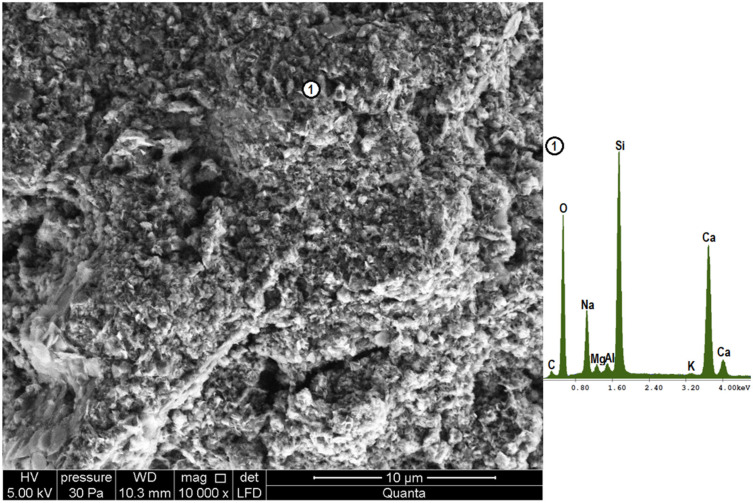
Microstructure of green glass powder after 330 days of storage in hydroxide solution (magnification 10,000×), together with the elemental composition analysis at point 1.

Figure 7, Figure 8 and Figure 9 show the microstructure of glasses stored for 330 days in hydroxide solution. In all cases, hydrated calcium silicates form upon reaction of glass powders with calcium hydroxide. For this reason, silicon and calcium are primarily identified in the elemental analysis. In addition to the resulting products, the silicon source can also be unreacted glass. Similarly, the source of calcium could be unreacted calcium hydroxide crystallized from the solution, but given the X-ray analysis carried out and the lack of observation of characteristic forms [4], this can be ruled out. As soda-lime glass was used in the study, it can also provide indications of both calcium and sodium. However, the high sodium readings are due to the presence of sodium hydroxide in the test solution.

In the case of the colorless glass, a loose, spongy microstructure composed of hydrated calcium silicates formed on contact with the hydroxide solution. They have an amorphous character typical of the C-S-H III phase in the Diamond classification based on morphological differences [4,48]. The observed C-S-H phase is composed of irregular, isometric particles less than 0.3 µm in size, forming dense clusters. A similar microstructure can also be observed in the brown glass sample (Figure 8 point 1). In addition to it, however, there are also hydrated calcium silicates with the structure of the so-called “honeycomb”, i.e., the C-S-H II phase, where the network of fibers or platelets forms a characteristic, three-dimensional structure. It is present under a layer of spongy phase, allowing it to be combined with interior products. This phase is characterized by a large number of unevenly distributed voids with a diameter of no more than 0.7 µm. This phase is characterized by a higher magnesium content than the previous phase. It can thus be combined with the saponite-like structures detected by X-ray analysis (Figure 3). The microstructure of samples with green glass powder is the most compact and homogeneous. It is made up, as with clear glass, of amorphous calcium silicates. The denser microstructure observed in the green glass sample aligns with Bahar et al. [32], who reported the lowest porosity for this type of glass. The extended formation time of hydrated calcium silicates (C-S-H) might explain this densification, similar to how cement hydrating with a setting retarder develops a more compact structure [4,49,50].

## 5. Conclusions

The research findings are as follows:

- Regardless of the color of the soda-lime glass, a pozzolanic reaction occurs when the glass powders come into contact with the calcium and sodium hydroxide solution, resulting in the formation of hydrated calcium silicates.
- The pozzolanic reaction of glass powders first produces hydrated calcium silicates structurally similar to tobermorite, later transforming into dellaite. It remains unclear whether dellaite-like structures result from the transformation of tobermorite-like products or form directly from the glass powder. Simultaneously, the background related to amorphous glass decreases, while that associated with amorphous calcium silicates increases.
- Higher levels of magnesium and iron oxides may lead to the formation of other products, such as saponite.
- Green glass, rich in chromium, reacts more slowly than clear or brown glass but forms a denser microstructure.
- For practical or performance evaluations, it could be beneficial to separate green glass from mixed cullet, due to its different characteristics.
- After 330 days, the complete pozzolanic reaction is confirmed by the total consumption of calcium hydroxide, as shown by XRD and SEM analyses.
- Additional detailed thermal studies are recommended to identify which decomposition phases produce maximum thermal effects at 125, 180, and 440 °C.

## Figures and Tables

**Table 1:** Chemical composition of different-color glass [36].

Oxide Glass	Colorless	Brown	Green
SiO_2_	72.53	71.56	72.11
Na_2_O	13.03	13.13	13.15
CaO	10.53	10.28	10.01
Al_2_O_3_	1.93	1.72	1.73
MgO	1.19	2.14	1.44
K_2_O	0.36	0.47	0.57
SO_3_	0.14	0.03	0.02
Fe_2_O_3_	0.08	0.41	0.43
TiO_2_	0.07	0.11	0.14
SrO	0.05	0.02	0.04
ZrO_2_	0.02	0.02	0.02
P_2_O_5_	0.01	0.01	0.01
Mn_2_O_3_	0.01	0.05	0.03
Cr_2_O_3_	0.00	0.04	0.25

**Table 2:** Phases present in the colorless, brown, and green glass samples as a function of time.

Time Phase	1 h	7 d	28 d	56 d	330 d
calcite					
				
				
portlandite					-
					-
					-
thermonatrite			-	-	-
			-	-	-
			-	-	-
tobermorite	-	-			
	-	-			
	-	-	-		
dellaite	-	-	-	-	
	-	-	-	-	
	-	-	-	-	
wollastonite	-	-	-	-	
	-	-	-	-	
	-	-	-	-	
saponite	-	-	-	-	-
	-	-	-	-	
	-	-	-	-	-
The presence of the phase in the sample:					
colorless glass		brown glass		green glass	

**Table 3:** Data from the thermal analysis (Figure 6) of glass powders treated with hydroxide solution for 28 days.

No	Temperature Range [°C]	Maximum [°C]	Loss for Colorless Glass [%]	Loss for Brown Glass [%]	Loss for Green Glass [%]
1	50–100	98	0.767	0.848	0.557
2	100–150	125	1.162	0.768	0.624
3	150–200	180	0.740	1.525	1.572
2 + 3	100–200	-	1.902	2.293	2.196
1 + 2 + 3	50–200	-	2.669	3.097	2.753
4	400–470	440	0.578	0.498	0.562
	Calculated Ca(OH)_2_		2.375	2.049	2.309
5	480-630	610	3.659	3.491	2.479
6	630-770	705	6.123	7.034	8.105
	Calculated CaCO_3_		13.925	15.997	18.433
	Amount of Ca(OH)_2_ needed to form CaCO_3_		10.309	11.843	13.646
5 + 6	480–770	-	9.782	10.525	10.584

## Data Availability

The raw data supporting the conclusions of this article will be made available by the authors on request.
